# A Flexible Wearable Data Glove Based on Hybrid Fiber-Optic Sensing for Hand Motion Monitoring

**DOI:** 10.3390/ma19081525

**Published:** 2026-04-10

**Authors:** Jing Li, Xiangting Hou, Ke Du, Huiying Piao, Cheng Li

**Affiliations:** 1 School of Smart City, Beijing Union University, Beijing 100101, China; 20252085410225@buu.edu.cn (X.H.); 20252085410223@buu.edu.cn (K.D.); 2 School of Instrumentation and Optoelectronic Engineering, Beihang University, Beijing 100191, China; sy2217323@buaa.edu.cn

**Keywords:** hybrid sensing, fiber Bragg grating, plastic optical fiber, wearable optical fiber data glove, hand motion monitoring

## Abstract

Wearable data gloves often suffer from electromagnetic interference, insufficient substrate stability, and limited capability for multi-degree-of-freedom motion measurement. To address these limitations, a flexible glove incorporating a hybrid POF-FBG sensing scheme was designed and fabricated. Plastic optical fibers (POFs) were side-polished and patterned with long-period gratings to improve sensitivity to wrist flexion-extension and abduction-adduction. Then fiber Bragg gratings (FBGs) were embedded in a polydimethylsiloxane substrate and encapsulated using thermoplastic polyurethane fixtures to reduce the influence of skin stretching and improve measurement accuracy of finger-joint angle. Moreover, a thermoplastic polyurethane skeleton with an adaptive sliding-rail structure was 3D printed to maintain the stability of the sensor placement at the joints. Experimental results demonstrated the mean absolute errors of 4.06°, 1.38° and 1.70° for wrist flexion-extension, abduction-adduction and finger-joint bending, respectively, along with excellent gesture classification using a support vector machine algorithm, which indicates great potential in virtual reality interaction and hand rehabilitation applications.

## 1. Introduction

Hand motion monitoring plays a critical role in emerging applications such as virtual reality (VR) and augmented reality (AR) interaction, hand rehabilitation, telemedicine, and human-computer interfaces [1,2,3]. The ability to accurately identify wrist and finger motions provides the basis for assessing hand function and implementing human-computer interaction.

Existing hand motion capture technologies can be broadly categorized into vision-based, inertial-based, and wearable sensor-based approaches. Vision-based systems provide contactless tracking but are highly sensitive to illumination variation and line-of-sight occlusion, which significantly limits their robustness in complex or unstructured environments [4]. Inertial measurement unit (IMU)-based gloves offer portability but inevitably suffer from integration drift and susceptibility to magnetic interference, leading to cumulative errors during long-term use [5]. Electronic skin and flexible sensor gloves based on resistive or capacitive transduction have also been extensively studied; however, these devices often exhibit hysteresis, nonlinear responses, and signal instability under repeated deformation [6,7,8].

Optical fiber sensors have attracted increasing attention in wearable hand motion monitoring due to their intrinsic immunity to electromagnetic interference, lightweight nature, and high sensitivity [9,10,11]. Among them, fiber Bragg grating (FBG) sensors are widely used for finger joint angle measurement owing to their high strain sensitivity and multiplexing capability [12,13,14,15]. Nevertheless, when subjected to large-amplitude wrist motions, FBG sensors may experience signal saturation, nonlinear response in high-strain regimes, or even mechanical failure due to excessive bending strain [16,17]. As a result, fully FBG-based data gloves are generally limited to small-range finger motions, with wrist flexion-extension ranges often constrained to within ±45° [18].

In contrast, plastic optical fiber (POF)-based sensors exhibit superior flexibility and can tolerate large bending deformation, making them suitable for monitoring large-amplitude wrist movements [19,20,21]. However, POF sensors typically rely on intensity modulation mechanisms and therefore suffer from relatively low sensitivity to small strain variations, which restricts their ability to resolve subtle finger joint motions with high precision [22]. Consequently, data gloves based on a single fiber sensing mechanism face inherent trade-offs between measurement range and sensitivity, limiting their ability to simultaneously capture multi-degree-of-freedom hand motions.

In addition to sensing limitations, mechanical integration remains a critical challenge for wearable fiber-optic gloves. Most existing designs directly stitch or embed optical fibers into textile substrates, which are highly extensible and prone to deformation during repeated wearing and doffing [23]. Relative displacement between the sensors and anatomical joints, as well as parasitic strain induced by skin stretching, can significantly degrade measurement repeatability and long-term stability. These issues become particularly pronounced during prolonged use, limiting the reliability of wearable hand motion monitoring systems.

To address the aforementioned challenges, this work proposes a flexible wearable data glove based on a hybrid fiber-optic sensing strategy. The primary contributions and novelties of this study are three-fold. Firstly, unlike conventional single-mechanism gloves or our previous single-joint POF sensing work [22], we implemented a heterogeneous sensing architecture that utilizes sensitivity-enhanced POF for large-range wrist motion and encapsulated FBG for high-precision finger-joint monitoring. This hybrid approach ensures both a wide measurement range and high angular resolution across 14 joints. Secondly, we designed a genuinely new rigid-flexible hybrid carrier—a 3D-printed thermoplastic polyurethane (TPU) skeleton with an adaptive sliding-rail structure. This mechanism effectively decouples the sensors from parasitic strain induced by skin stretching, significantly improving measurement repeatability. Thirdly, by integrating these heterogeneous sensors with a refined mechanical structure, the proposed system aims to achieve highly precise, multi-degree-of-freedom hand motion tracking and robust real-time gesture recognition.

## 2. Sensor Fabrication and Assembly

### 2.1. Sensing Principles

The hand motion capture is concerned with wrist and finger motion detections based on distinct optical sensing mechanisms. For wrist, the sensitivity of the POF to bending motion is enhanced through a combination of side-polishing and long-period grating (LPG) inscription. The side-polished D-shape structure breaks the total internal reflection condition at the interface, intensifying the evanescent field leakage. As the fiber is curved, the power loss coefficient increases following the modified bending loss model. The integration of an LPG further promotes mode coupling between the guided core modes and radiation modes through periodic geometric perturbations. This dual sensitization allows for high-sensitivity detection of wrist flexion and abduction across large angular ranges.

For finger joint, the FBG detects bending motion by converting it into axial strain. When a bare fiber is curved with a radius R, the resonance wavelength shift is minimal. By embedding the FBG into a PDMS (Polydimethylsiloxane) substrate at a distance x from the mechanical neutral axis, the bending-induced strain ϵ can be expressed as:

(1) $$\epsilon =\frac{x}{R}$$

Consequently, the Bragg wavelength shift Δλ is governed by:

(2) $$\Delta \lambda =\lambda \left(1-P_{e}\right)\frac{x}{R}$$

where $P_{e}$ is the effective photo-elastic coefficient (approximately 0.78). This encapsulation strategy ensures that finger bending is efficiently translated into a measurable spectral shift, while the high elasticity of PDMS and the 3D-printed fixture protect the fiber from mechanical failure during repetitive movements.

### 2.2. Design and Fabrication of Hybrid Optical Fiber Sensing Unit

**Figure 1:**
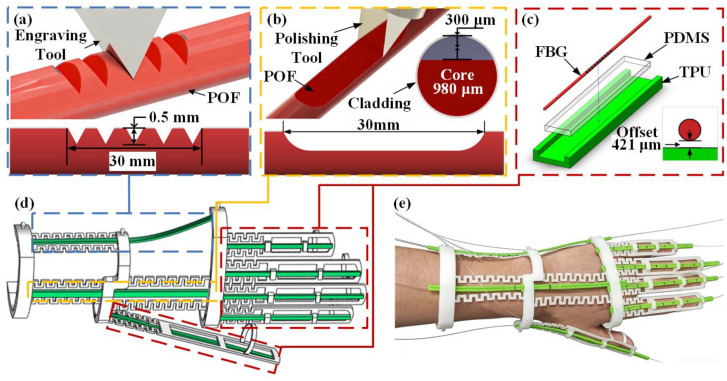
Design and fabrication of the hybrid sensing data glove. (**a**) Schematic of the long-period grating POF sensor (based on [22]). (**b**) Schematic of the side-polished POF sensor (based on [22]). (**c**) Exploded view of the FBG sensor encapsulation with neutral axis offset. (**d**) 3D model of the flexible glove skeleton. (**e**) Photograph of the prototype glove and real-time display interface.

To accommodate the substantially different deformation characteristics associated with wrist and finger motions, hybrid fiber sensing units were designed using distinct sensing mechanisms and structural configurations. In particular, plastic optical fiber (POF) sensors were employed for wrist motion monitoring, while fiber Bragg grating (FBG) sensors were adopted for fine finger joint sensing. The hybrid sensing system integrates two POF sensors and five optical fiber strings containing fourteen FBG sensing nodes for measuring the motions of wrist and finger joints, respectively. To capture the full range of hand movement, a two-node FBG string is assigned to the thumb, while the remaining four fingers are each equipped with a three-node string to monitor the MCP, PIP, and DIP joints. A step-index POF with a diameter of 1 mm (Mitsubishi ESKA-SK40, Tokyo, Japan) was used as the wrist sensing element. The design and surface-treatment fabrication process of these POF sensors build upon the methodology previously proposed by our research group [22]. To achieve high sensitivity for small-amplitude wrist motions, such as abduction and adduction, a periodic V-groove structure was mechanically inscribed on the POF surface, as shown in Figure 1a. The groove period was set to 3 mm, with a depth of 0.4 mm, and a total of ten grooves were fabricated along the sensing region. By introducing periodic geometric perturbations along the fiber surface, the V-groove structure amplifies microbending-induced optical power loss, resulting in improved sensitivity to small-angle bending. This configuration was therefore selected to capture subtle wrist motions within a limited deformation range while maintaining a compact sensing length. For large-amplitude wrist flexion and extension, a side-polished POF structure was fabricated using a customized 3D-printed mold. As illustrated in Figure 1b, a flat polished region with a length of 30 mm and a depth of 0.4 mm was formed on the fiber surface. When subjected to large bending deformation, the side-polished region significantly intensified optical leakage, enabling a stable and monotonic intensity response over a wide angular range. Compared with the grooved structure, the side-polished design provides a broader dynamic range, making it more suitable for large-angle wrist motion monitoring. To capture fine finger joint motions, a TPU-PDMS (Polydimethylsiloxane)-FBG composite encapsulation structure was employed, as depicted in Figure 1c. FBGs with the grating length of 10 mm and central wavelengths distributed between 1528 nm and 1568 nm were pre-tensioned and fixed within a mold. PDMS was then injected and cured at 60 °C for 60 min [18] to form a flexible substrate. The cured PDMS-FBG unit was subsequently embedded into a 3D-printed TPU fixture, which provided mechanical protection and ensured stable grating positioning during operation. This encapsulation strategy enables the FBG sensor to reliably resolve small bending-induced strain variations associated with finger joint motion [24].

### 2.3. Flexible Skeleton Fabrication and Glove System Assembly

To ensure consistent sensor performance during wear and hand motion, a flexible glove skeleton was fabricated via fused deposition modeling (FDM) using TPU (Shore hardness 95A). The skeleton served as a mechanical carrier for the heterogeneous sensing units while reducing the influence of skin stretching on sensor outputs. As shown in Figure 1d, the skeleton adopted a modular architecture comprising fingertip, phalangeal, palm, and wrist modules. These modules were interconnected by wave-shaped elastic joints, allowing the structure to accommodate variations in hand size and joint motion range. To further enhance sensor stability during joint movement, a miniature sliding-rail structure was integrated into the phalangeal modules. During assembly, one end of each FBG sensor was fixed at the proximal phalanx, while the other end was placed within the sliding rail. This configuration allowed limited relative sliding during joint bending, functioning as a structural decoupling mechanism that effectively isolates the FBG sensors from parasitic strain induced by skin stretching. For wrist motion sensing, the POF sensors were routed along the back and lateral sides of the wrist module, with their sensing regions securely anchored to the designated slots on the skeleton to capture large-amplitude wrist deformations. Through this structural arrangement, the different sensing units were mechanically integrated into a unified wearable system without introducing rigid constraints on natural hand movement. The fully assembled wearable data glove is shown in Figure 1e.

## 3. Experimental Results and Discussion

### 3.1. Experimental Setup

**Figure 2:**
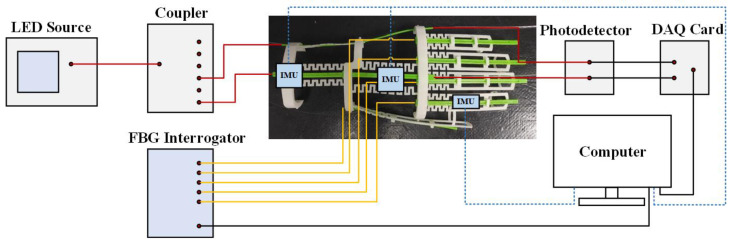
Schematic diagram of the experimental setup. The system comprises three distinct signal channels: the intensity-modulated POF sensing channel (red lines), the wavelength-modulated FBG sensing channel (yellow lines), and the IMU reference detection channel (blue dashed lines). All sensor data are synchronized and processed in the computer.

Figure 2 shows the connection architecture of the experimental test platform, wherein the entire system consists of two parallel and independent optical sensing channels, as well as one set of inertial reference detection channels. The part indicated by the red lines corresponds to the POF sensing channel, which employs a high-stability red LED light source with a wavelength of 650 nm as the input. The optical beam is split and distributed to the POF sensors mounted on the wrist of the glove via an optical coupler. The modulated output optical signal is received by a photodetector and converted into a voltage signal, which is ultimately transmitted to a computer through a data acquisition card. The part indicated by the yellow lines corresponds to the FBG sensing channel, which employs an FBG interrogator integrated with a broadband light source and a spectral analysis module. The interrogator is connected to the FBG sensor array mounted on the fingers of the glove via single-mode fiber patch cords, performing real-time demodulation of the central wavelength shifts. The signals are integrated into two distinct hardware channels. Specifically, the wrist POF signals are processed through an intensity-modulation channel where intensity variations are converted into voltage by photodetectors and captured by a USB-6212 DAQ card. Simultaneously, the finger FBG signals are resolved through a wavelength-modulation channel using an FI-108 interrogator, which employs five independent optical ports to simultaneously demodulate the center wavelength shifts from all nodes. The part indicated by the blue dashed lines in Figure 2 corresponds to the inertial reference detection channel, which incorporates a nine-axis IMU reference system composed of three nodes. To address the load sensitivity of finger joints, a heterogeneous layout strategy is adopted by use of two standard-sized IMUs and one miniature IMU. The former two standard-sized IMUs are fixed to the subject’s forearm and the back of the hand, respectively, for detecting wrist motion, while the latter miniature IMU is attached to the proximal phalanx of the index finger to reduce mechanical obstruction to finger joint movement. By synchronously collecting spatial orientation data from the three IMU devices attached to the forearm, the back of the hand and the proximal phalanx, the reference angles for sensor evaluation were acquired. For this study, the performance evaluation was conducted on one single healthy volunteer. The dynamic hand motion data used for model development (2764 samples) and independent validation (3781 samples) were collected in separate experimental sessions conducted on different days. Between these acquisitions, the glove was fully removed and re-worn, and the experimental setup was re-initialized before validation. A coordinate transformation algorithm based on rotation matrices was applied to calculate the bending angles of wrist and finger joints. The algorithm determines the relative orientation of the hand from the perspective of the forearm, thereby isolating physiological movements from absolute spatial postures. In three-dimensional space, coordinate transformation can be achieved through three successive rotations about the X-axes, Y-axes, and Z-axes, corresponding to pitch(φ), roll(θ), and yaw(ψ) angles, respectively. Since the Euler angles provided by the IMU follow the ZYX rotation sequence (yaw, roll, pitch), and therefore the rotation matrix $R_{321}\left(\psi ,\theta ,\varphi \right)$ is formulated as follows:

(3) $$R_{321}\left(\psi ,\theta ,\varphi \right)=R_{x}\left(\varphi \right)R_{y}\left(\theta \right)R_{z}\left(\psi \right)=\left[\begin{array}{ccc} \cos\theta \cos\psi & \cos\theta \sin\psi & -\sin\theta \\ \sin\varphi \sin\theta \cos\psi -\cos\varphi \sin\psi & \sin\varphi \sin\theta \sin\psi +\cos\varphi \cos\psi & \sin\varphi \cos\theta \\ \cos\varphi \sin\theta \cos\psi +\sin\varphi \sin\psi & \cos\varphi \sin\theta \sin\psi -\sin\varphi \cos\psi & \cos\varphi \cos\theta \end{array}\right]$$

Two IMUs are mounted on the hand back and forearm, whose coordinate systems are denoted as $C_{a}$ and $C_{b}$, respectively, while the global coordinate system is denoted as $C_{0}$. A direction vector $p_{a}=\left(0,1,0\right)$ is defined in $C_{a}$ to represent the palm direction. The vector $p_{a}$ is first transformed into the global coordinate system $C_{0}$ as ${p_{a}}^{\prime }={R_{a}}^{-1}p_{a}$, and then into the forearm coordinate system $C_{b}$ as ${p_{a}}^{\prime \prime }=R_{b}{p_{a}}^{\prime }$. Through this two-step transformation, the vector $p_{a}$ is expressed in a unified coordinate system.

Let ${p_{a}}^{\prime \prime }=\left(x,y,z\right)$, the wrist motion angles are calculated based on the geometric relationship of the vector components, where the flexion-extension is defined as α and abduction-adduction angle is defined as β, which can be extracted using fundamental inverse trigonometric functions:

(4) $$\alpha =\arctan\frac{z}{y}$$

(5) $$\beta =\arctan\frac{x}{y}$$

### 3.2. Sensor Calibration and Characteristic Analysis

**Figure 3:**
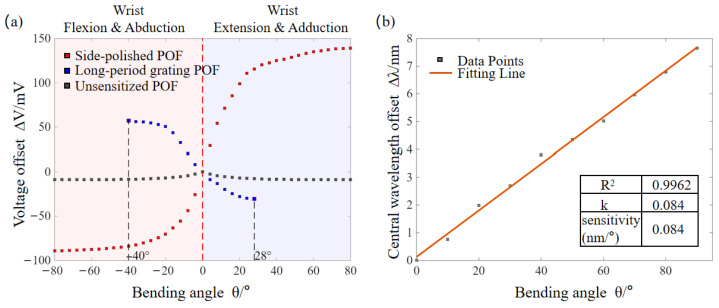
Calibration results of (**a**) the POF sensor for wrist bending and (**b**) the FBG sensor for finger bending.

Note that for determining the input-output characteristics of the plastic optical fiber (POF) and fiber Bragg grating (FBG) sensors, static calibration experiments were conducted on the two heterogeneous sensing units. In this process, the raw output voltages were referenced to the baseline value at the neutral position (0°) to obtain a normalized voltage offset. The detailed mathematical normalization procedure and the definitions of $V_{1}$ and $V_{2}$ are provided in Section 3.3 to support the subsequent dynamic tracking analysis. For wrist motion monitoring, to test the sensitization effect of the designed POF sensor and select the one with optimal sensitivity to bending, Figure 3a shows the voltage response characteristics of three types of POF sensors with different structures, namely side-polished, grating, and unsensitized ones, within their respective operating ranges. Specifically, the output voltages were recorded at 4° intervals for the side-polished POF over the wrist flexion-extension range of −80° to +80°, the long-period grating POF over the abduction-adduction range of −40° to +28°, and the unsensitized POF serving as the control group. The experimental results show that the normalized voltage of the three types of POF sensors exhibits a nonlinear relationship with the bending angle. Benefiting from the effective regulation of the evanescent field by its D-shaped structure, the side-polished POF shows the most significant voltage variation, thus achieving the highest sensitivity. For knuckle motion monitoring, Figure 3b shows the central wavelength response of the PDMS-encapsulated FBG sensor over the range of 0° to 90°. The experiment recorded the central wavelength at 10° intervals as the joint angle varied within this range. The results reveal that the central wavelength shift (Δ*λ*) of the FBG exhibits extremely high linearity with the finger joint bending angle (*θ*) (goodness of fit *R*^2^ = 0.9962), with a sensitivity approximated as 0.084 nm/°. This result strongly confirms that the encapsulation structure of the TPU fixture not only effectively protects the grating, but also provides a stable strain transmission path, which efficiently converts joint bending into axial strain in the optical fiber and thus satisfies the requirements for high-precision detection of minute deformations of finger joints.

### 3.3. Dynamic Tracking Performance and Comparative Analysis

For wrist motions, the output voltage of the POF sensors and the IMU reference data were synchronously collected. To eliminate differences in initial optical power among individual POF sensors, the instantaneous output voltage ($V_{i}$) of the respective POF sensor was normalized. To account for the bidirectional movement of the wrist from the neutral position, a piecewise normalization approach was adopted. Using the reference voltage at 0° ($V_{0}$) as the baseline, along with the maximum ($V_{\max}$) and minimum ($V_{\min}$) voltages recorded during calibration, the normalized voltages were derived. Specifically, $V_{1}$ and $V_{2}$, respectively, represent the normalized voltages for the positive bending direction (wrist extension or abduction) and the negative bending direction (wrist flexion or adduction):

(6) $$V_{1}=\frac{V_{i}-V_{0}}{V_{\max}-V_{0}}$$

(7) $$V_{2}=\frac{V_{i}-V_{0}}{V_{0}-V_{\min}}$$

**Figure 4:**
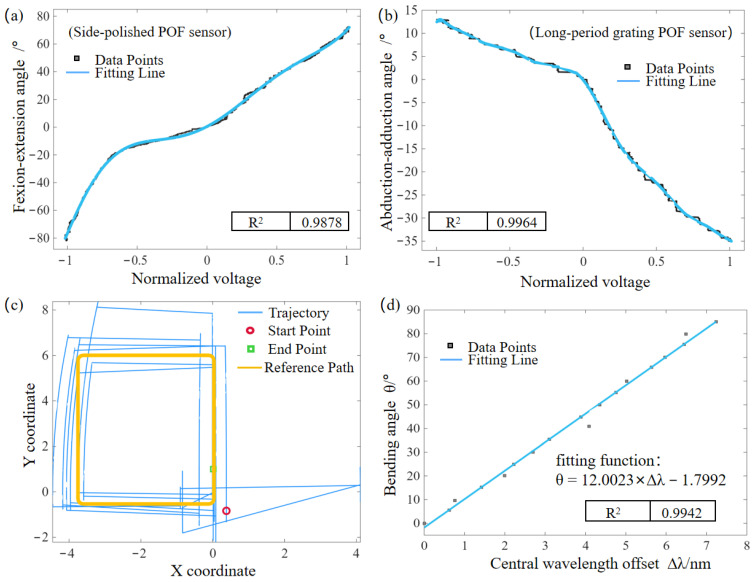
Experimental results of dynamic tracking and calibration. (**a**) Fitting curve of the wrist flexion-extension angle versus the normalized voltage of the side-polished POF sensor. (**b**) Fitting curve of the wrist abduction-adduction angle versus the normalized voltage of the long-period grating POF sensor. (**c**) Reconstructed 2D trajectory of the wrist motion forming a rectangular path. (**d**) Linear fitting relationship between the finger joint bending angle and the central wavelength shift of the FBG sensor.

By mathematically combining $V_{1}$, ranging from 0 to 1, and $V_{2}$, ranging from −1 to 0, a complete normalized voltage spanning from −1 to 1 was established for each POF sensor. Then the normalized voltages were substituted into the polynomial fitting functions (obtained during calibration, as shown in Figure 4a,b) to calculate the estimated bending angles for wrist flexion-extension and abduction-adduction movements. These derived angles were then compared with the reference angle values from the IMU devices. The mean absolute errors (MAEs) were calculated as 4.06° and 1.38° for flexion-extension and abduction-adduction motions, respectively. The comparison of the two MAEs reveals that the slightly larger error (4.06°) in palmar flexion-extension is primarily due to the motion range of this degree of freedom exceeding 150°. This resulting large angular range would impose excessive bending deformation on the side-polished POF sensor and exacerbate the nonlinearity of its intensity-modulated response to bending strain. The increased measurement error is further verified to be associated with minor sensor displacements induced by skin folds at the wrist joint during large-amplitude flexion-extension movements, which introduces unintended optical power loss and thus interferes with the stable detection of POF light intensity signals.

Furthermore, the derived angles were applied to a wrist motion trajectory tracking experiment so as to evaluate the dynamic 2D tracking capability of the glove. Based on the palm length *L*, the derived flexion-extension angle *α*, and the abduction-adduction angle *β*, the trajectory coordinates (*x*,*y*) were calculated using the kinematic model:

(8) x=Lcosαsinβ

(9) y=Lcosαcosβ

As shown in Figure 4c, the reconstructed trajectory approximates a closed rectangular loop, validating the sensor’s ability to decouple complex 2D motions.

Then for finger joint motion, dynamic calibration experiments were conducted within the range of 0° to 85°, with data recorded at intervals of 5° to obtain the fitting function in Figure 4d. In the experiments, a miniature IMU was employed to record the actual joint bending angles, while an FBG interrogator was simultaneously used to collect the corresponding central wavelength shifts. New wavelength and angular data were subsequently acquired independently; the wavelength data were substituted into the fitting function to calculate the estimated angles, which were then compared with the newly measured actual bending angles of the finger joints. The mean measurement error of the finger joint angles was calculated as only 1.70°.

To provide a clearer comparison with representative state-of-the-art wearable hand sensing systems, Table 1 summarizes several prior works in terms of sensing principle, monitored joints, measurement range, reported accuracy, and gesture recognition capability. The selected studies cover different sensing paradigms, including FBG-based and POF-based sensing approaches. As shown in Table 1, FBG-based systems generally achieve high angular accuracy but are often limited to finger motion tracking and lack gesture recognition capability. POF-based systems provide a wider measurement range for wrist motion but typically exhibit moderate accuracy. Flexible strain-based systems enable gesture recognition but do not provide precise joint angle measurements. In contrast, the proposed hybrid POF–FBG sensing strategy combines the advantages of both sensing mechanisms, thus enabling multi-joint finger motion tracking with relatively high accuracy while maintaining a wide measurement range for wrist movement. Furthermore, the developed glove system supports gesture recognition, achieving a balanced performance in terms of accuracy, range, and functionality.

Table 1 compares the performance between the proposed glove and representative wearable hand sensing systems. The glove system in this work utilizes wavelength multiplexing to monitor fourteen nodes using five fiber strings, which reduces wiring complexity and improves wearability. In addition, the 3D-printed modular skeleton with integrated mounting slots simplifies assembly and supports cost-effective fabrication. Furthermore, the integration of multiple sensing nodes enables expanded sensing coverage without significantly increasing wiring complexity, providing good scalability for wearable applications. These advantages make the hybrid sensing glove a greatly potential solution for large-scale applications in rehabilitation and virtual reality.

The repeatability and structural stability are critical factors for the practical deployment of the data glove. In this study, the consistent performance across independent experimental sessions, involving complete wearing and doffing cycles, demonstrates the short-term repeatability of the integrated system. Furthermore, the use of high-resilience elastomers (TPU and PDMS) combined with the sliding-rail mechanism is designed to reduce cumulative mechanical fatigue on the sensors. However, comprehensive quantitative evaluations of its long-term durability over extensive life cycles remain to be systematically addressed in future studies.

### 3.4. Gesture Recognition Experiments

**Figure 5:**
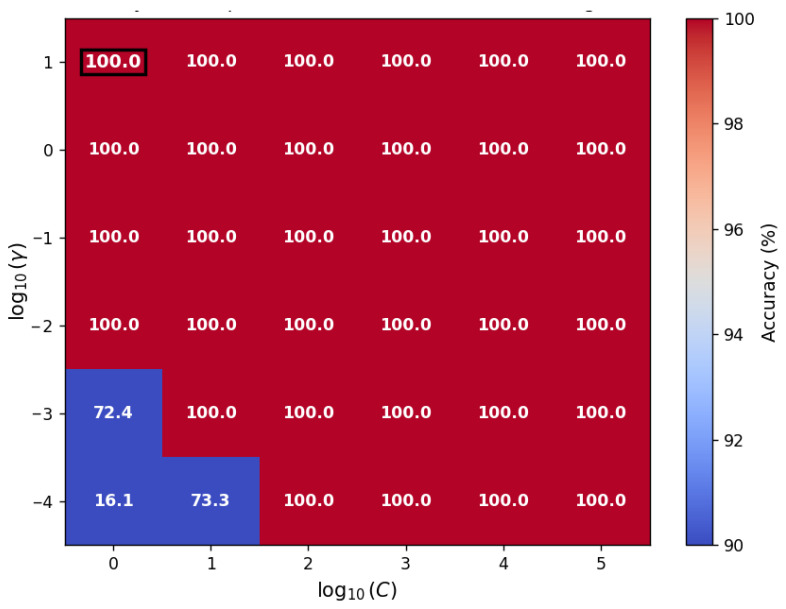
Heatmap of classification accuracy of the RBF-SVM model as a function of the penalty factor C and kernel parameter γ, obtained via grid search with five-fold cross-validation. The optimal parameter combination (C=0.1 and γ=10) is highlighted with a black square.

Furthermore, to evaluate the glove system’s performance in real-world interactions, gesture recognition experiments were conducted. Fourteen FBG sensing nodes were distributed across the hand to capture the motion of the metacarpophalangeal (MCP) and proximal interphalangeal joints of the five fingers (two nodes for the thumb and three for each of the other four fingers). Based on the wavelength shifts of these FBGs, seven typical gestures corresponding to digits 1 to 7 were defined. Then a total of 2764 sets of labeled data were initially collected. This primary dataset was randomly divided into a training set and a test set with a ratio of 7:3. A Support Vector Machine (SVM) algorithm with a radial basis function kernel was employed to train the classification model. Moreover, an additional independent dataset comprising 3781 samples was collected to construct a validation set for further examining the model’s generalization capability and robustness (beyond the initial train-test split). To optimize the classification performance, multiple kernel functions, including linear, polynomial, sigmoid, we first compared various kernel functions. The RBF kernel was selected for its superior capability in handling non-linear relationships, as supported by established literature [27]. Subsequently, a grid search with five-fold cross-validation was performed to tune the RBF-SVM hyperparameters (C and γ). The results, presented in Figure 5, show that the model achieves 100% accuracy across a broad parameter range, indicating that our hardware-acquired data possess high discriminative features and are robust against hyperparameter selection. Thus, C=0.1 and γ=10 were confirmed for the final implementation to balance model simplicity and generalization capability.

To evaluate the model’s reliability under real-world conditions, the 3781 samples in the independent validation set were collected in a separate session from the initial training data. Specifically, the participant was required to remove and re-wear the glove, and the hardware interface was reset prior to validation. The achievement of 100% accuracy on this independent set demonstrates the cross-session stability of the sensing features, which is ensured by the mechanical stabilization provided by the adaptive sliding-rail skeleton.

**Figure 6:**
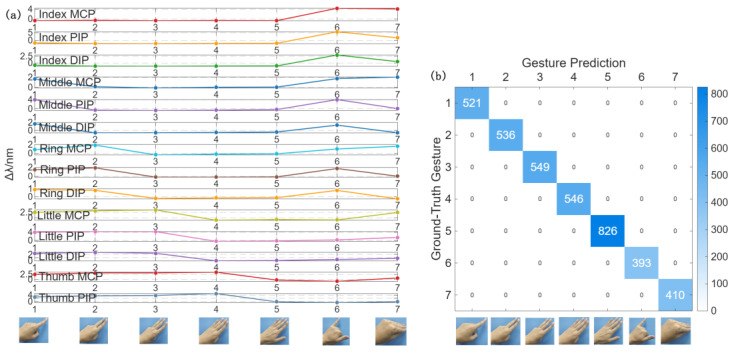
Gesture Recognition Results: (**a**) Center wavelength shifts corresponding to the calibrated positions of 7 gestures. (**b**) Confusion matrix of the validation set for 7 gestures.

Figure 6a illustrates the distinct center wavelength shift patterns of the used FBGs across the defined 7 gestures, displaying high feature separability. As shown in the confusion matrix in Figure 6b, the system demonstrated excellent performance on the validation set. Quantitative evaluation metrics, including recognition accuracy, precision, and recall, all reached 100%. This is visually confirmed by the diagonal dominance in the confusion matrix, where all off-diagonal elements are zero, indicating that no misclassification occurred among the seven gesture classes. Such high recognition rates are primarily attributed to the proposed hybrid sensing strategy. That is to say, the high-density FBG array and the flexible sliding-rail skeleton ensure precise capture of finger flexions, while the dual POF sensors concurrently monitor large-range wrist motion (as verified in Section 3.3), thus enabling the system to capture comprehensive hand motion information. This developed gesture recognition function can be flexibly adapted to practical needs such as gesture control in VR interaction, motion standard evaluation in hand rehabilitation training and doctor-patient gesture communication in telemedicine scenarios.

## 4. Conclusions

This paper implements a flexible wearable data glove based on a hybrid fiber-optic sensing strategy combining POF and FBG sensors. Specifically, side-polished and long-period V-groove POF structures are utilized to achieve high-performance detection of large dynamic range wrist movements, with mean errors of 4.06° and 1.38° for palmar flexion-extension and abduction-adduction degrees of freedom, respectively. Meanwhile, fourteen PDMS-encapsulated FBG sensors distributed across the MCP and interphalangeal joints of all five fingers enable fine detection of finger joint flexions, with an average error of 1.70°. Moreover, an SVM-based classification model was trained using 2764 datasets (with a 7:3 training-testing split), and an additional independent validation set comprising 3781 datasets was used to further evaluate the model generalization, which all demonstrated the recognition accuracy, precision and recall rate of 100% on the validation set. Although the current system demonstrates high precision and preliminary cross-session signal stability for one single healthy volunteer, we acknowledge several practical performance limitations. The long-term mechanical durability, hysteresis, and structural stability of the 3D-printed skeleton have not been quantitatively evaluated. Additionally, while the modular design is conceptually adjustable, its sensitivity to hand size differences and inter-user variability remains to be experimentally validated. Future work will focus on conducting rigorous mechanical durability tests and expanding the subject pool to include diverse hand sizes, thereby systematically evaluating the long-term robustness and cross-subject generalization of the hybrid sensing glove. Notably, an adaptive sliding-rail skeleton based on 3D-printed TPU was fabricated to physically decouple interference caused by skin stretching, thereby improving measurement repeatability and wearing stability during dynamic hand movements. Through the integration of hybrid sensing mechanisms and the adaptive sliding-rail structure, the developed glove system effectively offers the advantages of low-cost, electromagnetic interference immunity, range-accuracy balance and wearing stability for potential applications including virtual reality interaction, hand rehabilitation, and telemedicine. It should be noted that the influence of temperature fluctuations on FBG sensor response is considered negligible in this study due to the stable indoor environment of the intended applications such as virtual reality interaction and clinical rehabilitation. For future deployment in environments with changing temperatures, an additional FBG node can be integrated into each optical fiber string for temperature monitoring, and a standard temperature differential compensation scheme can be used to eliminate the thermal effect, along with the use of low-temperature drift material as the substrate structure installing FBG strings.

## Figures and Tables

**Table 1:** Comparison between the proposed glove and representative wearable hand sensing systems.

Method	Configuration	Performance		Dynamic Tracking	Gesture Recognition	Ref.
		Range	Accuracy			
FBG for finger and wrist movements	1 FBG node per joint, 14 nodes in total	0~110°	0.21° (mean)	Yes	No	[12]
FBG for finger movements	Multiple FBG nodes per joint, 39 nodes in total	— *	4.6° (maximum) 0.47 ± 2.51° (mean)	Yes	No	[14]
FBG for finger movements	1 FBG node per joint, 14 nodes in total	0~100°	0.176° (minimum)	No	Yes	[15]
POF for wrist movements	2 POF sensors at different wrist positions	−40~+40°	1.56° (flexion-extension, mean) & 2.94° (abduction-adduction, mean)	Yes	No	[22]
FBG for finger movements	1 FBG node per finger, 5 nodes in total	−40~+40°	2.9° (maximum)	Yes	No	[25]
FBG for finger movements	2 FBG node per finger, 10 nodes in total	0~80°	0.80° (mean)	No	No	[26]
POF for wrist movements + FBG for finger movements	Multiple FBG nodes per joint, 14 nodes in total	−81.6~+72.4° (wrist) 0~85° (finger)	4.06° (flexion-extension, mean) & 1.38° (abduction-adduction, mean) 1.70° (finger, mean)	Yes	Yes	This work

* Note: “—” indicates that the specific metric was not explicitly reported in the corresponding literature.

## Data Availability

The original contributions presented in this study are included in the article. Further inquiries can be directed to the corresponding authors.

## Abbreviations

The following abbreviations are used in this manuscript:

POF	Plastic Optical Fiber
FBG	Fiber Bragg Grating
TPU	Thermoplastic Polyurethane
PDMS	Polydimethylsiloxane
DOF	Degree of Freedom
MCP	Metacarpophalangeal Joint
DIP	Distal Interphalangeal Joint
PIP	Proximal Interphalangeal Joint
RBF	Radial Basis Function
SVM	Support Vector Machine
MAE	Mean Absolute Error
IMU	Inertial Measurement Unit
FDM	Fused Deposition Modeling
MCP	Metacarpophalangeal
